# Systematic evaluation of immune regulation and modulation

**DOI:** 10.1186/s40425-017-0223-8

**Published:** 2017-03-21

**Authors:** David F. Stroncek, Lisa H. Butterfield, Michael A. Cannarile, Madhav V. Dhodapkar, Tim F. Greten, Jean Charles Grivel, David R. Kaufman, Heidi H. Kong, Firouzeh Korangy, Peter P. Lee, Francesco Marincola, Sergio Rutella, Janet C. Siebert, Giorgio Trinchieri, Barbara Seliger

**Affiliations:** 1 Department of Transfusion Medicine, National Institutes of Health, 10 Center Drive, Building 10, Room 3C720, Bethesda, MD 20892 USA; 2 Department of Medicine, Surgery and Immunology, University of Pittsburgh Cancer Institute, 5117 Centre Avenue, Pittsburgh, PA 15213 USA; 3 Roche Pharmaceutical Research and Early Development, Roche Innovation Center Munich, Nonnenwald 2, 82377 Penzberg, Germany; 4 Department of Hematology & Immunobiology, Yale University, 333 Cedar Street, Box 208021, New Haven, CT 06510 USA; 5 GI-Malignancy Section, Thoracic and GI Oncology Branch, Center for Cancer Research, National Cancer Institute, National Institutes of Health, Building 10 Room 12 N226, 9000 Rockville, Bethesda, MD 20892 USA; 6 Division of Translational Medicine, Sidra Medical and Research Center, PO Box 26999, Al Luqta Street, Doha, Qatar; 7 Merck Research Laboratories, PO Box 1000, UG 3CD28, North Wales, PA 19454 USA; 8 Dermatology Branch, Center for Cancer Research, National Cancer Institute, National Institutes of Health, Building 10, MSC 1908, Bethesda, MD 20892-1908 USA; 9 Department of Immuno-Oncology, City of Hope, 1500 East Duarte Road, Duarte, CA 91010 USA; 10 The John van Geest Cancer Research Centre, Nottingham Trent University, Clifton Campus, Nottingham, NG11 8NS UK; 11 CytoAnalytics, 3500 South Albion Street, Cherry Hills Village, CO 80113 USA; 12 Cancer and Inflammation Program, Center for Cancer Research, National Cancer Institute, National Institutes of Health, Building 37/Room 4146, Bethesda, MD 20892 USA; 13 Institute of Medical Immunology, Martin Luther University Halle-Wittenberg, Magdeburger Str. 2, Halle, Germany

**Keywords:** Immune biomarkers, Systematic monitoring, Immune regulation, High-throughput

## Abstract

Cancer immunotherapies are showing promising clinical results in a variety of malignancies. Monitoring the immune as well as the tumor response following these therapies has led to significant advancements in the field. Moreover, the identification and assessment of both predictive and prognostic biomarkers has become a key component to advancing these therapies. Thus, it is critical to develop systematic approaches to monitor the immune response and to interpret the data obtained from these assays. In order to address these issues and make recommendations to the field, the Society for Immunotherapy of Cancer reconvened the Immune Biomarkers Task Force. As a part of this Task Force, Working Group 3 (WG3) consisting of multidisciplinary experts from industry, academia, and government focused on the systematic assessment of immune regulation and modulation. In this review, the tumor microenvironment, microbiome, bone marrow, and adoptively transferred T cells will be used as examples to discuss the type and timing of sample collection. In addition, potential types of measurements, assays, and analyses will be discussed for each sample. Specifically, these recommendations will focus on the unique collection and assay requirements for the analysis of various samples as well as the high-throughput assays to evaluate potential biomarkers.

## Background

Cancer immunotherapies such as immune checkpoint blockade, adoptively transferred T cells and natural killer (NK) cells, as well as antibody-based interventions and anti-tumor vaccination, are showing promising clinical results across a variety of malignancies [1]. Monitoring the immune response as well as tumor responses following these therapies has been important to the advancement of this field, and the identification of predictive biomarkers as well as early markers of response to new treatments are important goals of ongoing research in order to broaden the impact of these therapeutics. The validation of biomarkers predictive of treatment outcomes is paramount to identifying the patients who are most likely to benefit from treatment and/or to provide an early indication of therapy response (a topic addressed by WG1). At present no definitive biomarkers have been identified that can be used to predict which patients are most likely to have a clinical benefit. In melanoma, several preliminary biomarkers have been investigated in response to ipilimumab (anti-CTLA-4) treatment, but none have been validated in subsequent studies [2–5].

Peripheral blood is a readily (and repeatedly) accessible compartment that can yield valuable prognostic information, but the relationship between local immune responses within the tumor microenvironment (TME) and the peripheral immune system remains incompletely understood. Emerging data show that cancer and immune cells may be phenotypically and functionally different between primary tumors and metastatic tissues [6, 7]. Thus, it is often essential to monitor additional tissues to understand the impact of different immunotherapies on the host immune response. For example, tumor draining lymph nodes (TDLN) represent both a metastatic site as well as a major site of cancer-immune interactions [8, 9], the bone marrow is the dominant site of tumor involvement in several hematologic malignancies, and the cross-talk between the host and the commensal microbiome regulates many physiological functions including inflammation and immunity [10–12]. In addition, for adoptive cell therapies, it is important to analyze the administered cells as well as their persistence and trafficking in vivo.

It is critical to develop systematic approaches to monitor immune responses and to interpret the data obtained as the number of compartments and potential biomarkers analyzed increases. Biologic samples can now be analyzed at the cellular, DNA, transcriptional, epigenetic, post-transcriptional and protein levels, and the analysis of multiple compartments at several levels yields massive quantities of data, which require the use of novel analytic bioinformatics methods. The purpose of this review is to describe systematic approaches to monitor immune responses to cancer immunotherapy. Using blood, the TME, microbiome, bone marrow (BM) and transferred T cells as examples, the nature and timing of the samples that should be collected will be discussed as well as the potential types of measures, assays, and analyses. In particular, the unique collection and requirements for the analysis of blood and tissue and high-throughput assays suitable for evaluating these measures will be described.

## Monitoring a study

The advent and implementation of high-throughput technologies has made personalized, targeted tumor immunotherapy possible. In the development of cancer immunotherapies, the majority of work was done to identify proteins that are either overexpressed or mutated in patients’ cancer and could serve as the basis for a vaccine or of an adoptive immunotherapy. In the future, an individual patients’ pattern of serum antibody binding might be also used for the development of personalized immunotherapy as well as for monitoring immune responses. In addition, combinations of multiple high-throughput or “omics” technologies might help to identify these biomarkers. Predictive biomarkers are also required to link immunity with an increased likelihood of improved outcome for patients undergoing different immunotherapies. Often, the clinical efficacy of immunotherapies determined by anti-tumor responses has been associated with Th1 immunity [13].

For the monitoring of immune cell responses and tumor assessment using immunological markers, peripheral blood (peripheral blood mononuclear cells [PBMC] and serum) should be collected at baseline, early, middle, and late time points after onset of treatment with a follow-up after end of treatment again at early, middle, and late time points. In addition to conventional clinical lab analysis of lactate dehydrogenase, C-reactive protein (CRP), absolute lymphocyte count (ALC), immune cell repertoire (see flow cytometry), the expression of genes and proteins should be analyzed in serum/plasma for cytokines, chemokines, putative tumor-associated antigens and antibodies at the end of dosing and beyond [14]. Emerging studies also suggest the collection and analysis of tissues, bone marrow (particularly in hematologic malignancies) and microbiome [15–17].

Immunotherapies have become a standard treatment for some cancer types. The development and optimization of cancer immunotherapies to increase their efficacy have become intensive areas of research. Importantly, the identification of immune-related biomarkers for diagnosis, prognosis, monitoring of immune responses and identification of their mechanism of action, as well as for the selection of patients undergoing cancer immunotherapies and the prediction of clinical outcomes are also under intense investigation. The integration of multiple high-throughput “omics” technologies, including DNA sequencing, genome wide association studies, which allow for the identification of single nucleotide polymorphisms (SNP), and gene expression profiling of mRNA for the analysis of tumor or PBMC have been used to define such biomarkers. Furthermore, different proteome-based technologies, such as the serological evaluation of proteins and antibodies, top-down and bottom-up proteomics, multiparameter enzyme-linked immunosorbent assay (ELISA), and Luminex analyses have been employed for diagnosis, immune monitoring, immune response assays, and identification of novel therapeutic targets. The “ome”-based methods currently available have some advantages and disadvantages, such as sensitivity, reproducibility, amount of sample required for analysis, and that they strongly depend on the data analysis performed.

## Materials to be evaluated

An important issue for the development of high-throughput technologies related to cancer immunotherapies is the tissue source, with preference for easily accessible material, such as body fluids (blood and urine) rather than serial tumor biopsies, which are possible for cutaneous melanoma and hematologic malignancies, and more challenging for other tumor types where core biopsies (if anything) are more common. In this context, the capacity of the technology, the reproducibility of results, the assay stability and the ability to validate the results are essential considerations.

Sample generation, isolation, and processing are important issues, since significant differences have been observed between different methods and consumables used for the purification of serum, plasma, and immune cells obtained from peripheral blood and from tumor tissues [18–20]. In addition, the sample holding times before processing (1 – 48 h.), the blood collection method, and immunoglobulin G (IgG) purification from these samples could affect analysis, leading to reproducibility problems [21].

### Serum and plasma

Serum and plasma samples prepared from peripheral blood are easily obtainable from patients and are often collected as a part of clinical studies and stored in biobanks. In particular, serum or plasma is collected for the evaluation of cytokines, chemokines, and growth factors, as well as other soluble molecules, including antibodies, matrix metalloproteinases (MMP), and adhesion molecules [22, 23]. In addition, the newly appreciated role of exosomes and extracellular vesicles (EV) as cancer biomarkers [24] and in immune surveillance [25], begs for the development of sample collection methods compatible with multiple downstream analyses, including that of exosomes/microvesicles. The considerations linked to the choice of the source (whole blood versus plasma or serum), and the method of purification have been discussed in a position paper from the International Society of Extracellular Vesicles [26], which concluded that plasma is the most physiological relevant milieu to study blood EV. If such studies are envisioned, then plasma should be collected.

Serum samples can be collected using silica-coated serum separating tubes. Serum can then be incubated at a dilution of 1:50 in 0.5% casein-PBS (phosphate buffered saline) blocking buffer to suppress non-specific binding of sera proteins. For Luminex and ELISA, plasma samples can be collected in tubes containing one of three distinct anti-coagulants: (i) sodium heparin, (ii) sodium citrate dextrose and (iii) ethylenediaminetetraacetic acid (EDTA) [27].

To determine the serum peptidome profile, three different protocols can be used for mass spectrometric analyses of serum and plasma proteins. For the first, crude plasma samples can be directly subjected to tryptic cleavage. Otherwise, buffer components can be removed from the samples and samples can be concentrated using macro spin plates. For the third protocol, the process of depletion can be carried out using a proteoprep immunoaffinity albumin and IgG depletion kit, followed by trypsinization and peptide extraction on macro spin centrifuge plates. The protein digestion can be performed using trypsin at 37 °C for three hours with stirring. The trypsinization is then terminated by adding trichloroacetic acid, the pH adjusted, the trypsinized plasma is dried, resolved in liquid chromatography solution, spiked with isotypically labeled peptide standard and then used for mass spectrometric analysis [28]. Sample collection and preparation are critical steps to obtain useful information in clinical proteomics analyses. To circumvent undesirable degradation of proteins and peptides, serum samples should be collected under specific standard operating procedures (SOP). However, the current protocols and guidelines for human bodily fluid collection and storage prior to proteomic analysis, in particular regarding blood plasma and serum, still need to be optimized. The influence of pre-analytical factors on the serum peptidome profile is significant, especially the type of blood collection tube, variations in clotting time and temperature, storage conditions, and the number of freeze and thaw cycles [29–32].

Briefly, all venous blood specimens should be collected with vacuum blood collection tubes. After standing upright at room temperature for 60 min, the serum fraction is separated by centrifugation at 1500 x g for 15 min (4 °C) and immediately stored at -80 °C. Only one freeze and thaw procedure can be permitted for any serum sample used for mass spectrometric analysis (this is also critical for other assessments by approaches, such as Luminex because analytes are differentially sensitive to freeze/thaw cycles). The selection of the preservatives and additives used in the collection of blood is important in determining future applicability of the samples. For example, the collection of whole blood in tubes containing any type of anti-coagulant may induce cytokine production in vitro and thus results in artificial measures. Some coagulants are recommended or even required for particular analytical purposes, while others might be contraindicated [33].

The samples should be collected prior to treatment (baseline) and at various time points (e.g., early, middle, and late depending on the treatment interval) during therapy as well as after therapy (early, middle, and late time points). The samples should be aliquoted prior to freezing.

### Leukocytes

Complex immunoregulatory circuits, including the low frequency and activity of effector cells and high frequency of suppressive cells, have the potential to dampen the efficacy of immune interventions, thus cellular immune assessments should be considered an essential component of monitoring efforts in cancer immunotherapy clinical trials. Immune monitoring of peripheral blood and tumor immune cell infiltration offers insights into the mechanism(s) of action of immunotherapeutic strategies and may be prognostic of outcome. However, the selection of the methods and components analyzed during cellular monitoring of clinical trials clearly depends on the individual therapeutic modality and disease being investigated.

For these analyses, PBMC obtained from fresh anticoagulated whole blood are isolated by gradient centrifugation using ficoll or Histopaque^®^. Platelets are removed and any remaining contaminating red cells can be eliminated with ammonium chloride potassium (ACK) lysing buffer prior to the use of the cells for downstream analyses, e.g., flow cytometry, transcriptomics, and proteomics. It is noteworthy that hemolysis during sample preparation could significantly affect the biomarker content of e.g. cytokines, microRNA (miRNA) [34].

#### Leukocyte counts

Recently, studies have indicated that early changes in immunological markers may be associated with improved survival. To date, many of these signals have come from single analyte measures tested in some trials and not others, or from common clinical laboratory tests. Increases in ALC and eosinophil count after treatment with ipilimumab 3 mg/kg both correlated with improved survival [35]. Additionally, among 27 patients treated with ipilimumab 10 mg/kg, changes in the number of circulating T cells that expressed ICOS during the early treatment stages and a low ratio between absolute neutrophil count and ALC were also associated with better survival [36]. This is consistent with other analyses of patients treated in the expanded access program, where a high ALC after two doses of ipilimumab or at 6 weeks was significantly associated with survival [4, 37]. The association of changes in ALC with survival was also recently assessed among approximately 2000 patients who had received ipilimumab (at various doses as a monotherapy or in combination with chemotherapy) as part of their treatment regimen. Consistent with its proposed mechanism of action, treatment with ipilimumab resulted in an increase in mean ALC. However, while a positive association was observed between the rate of increase in ALC and survival, absolute changes in ALC were not found to be specifically predictive of improved survival [38]. By contrast, Simeone and co-authors showed that an increase in ALC between baseline and week 12 was significantly associated with disease control and survival in patients treated with intravenous ipilimumab 3 mg/kg every 3 weeks for a total of four doses [14]. Since ALC is a single analyte, further investigations into the utility of ALC as a prognostic biomarker of response to new drug activity are warranted, and it is suggested to combine ALC with other candidate markers.

#### T cells

It is now established that the infiltration of tumors by T cells can affect tumor growth, invasion, and patient outcome. Several studies have highlighted the correlation between ALC and clinical outcome both in patients with hematological malignancies and in those with solid tumors [39–41]. A conspicuous (“brisk”) lymphocyte infiltrate correlates strongly with a positive outcome in melanoma and in colorectal cancers (CRC). A follow-up study of 2845 patients with invasive primary melanoma has shown that death as a result of melanoma was 30% less with non-brisk tumor infiltrating lymphocyte (TIL) grade and 50% less with brisk TIL grade when compared with the absence of TIL independently of tumor characteristics currently used to define the melanoma stage [42]. In general, TIL express a CD3+CD8+CD45RO+ phenotype [43].

Numbers of CD8+ T cells correlate with improved outcome in various tumor types, including lung cancer and CRC [44, 45]. In contrast, tumor-infiltrating CD4+ T cell numbers may portend both favorable and unfavorable implications for patients’ survival. Regulatory T cells (Treg) express CD4 and reportedly constitute 5-15% of infiltrating CD4+ T cells in tumor samples [46]. The ratio of CD8+ T cells to Treg in TIL has been correlated with aggressive growth and poor response to chemotherapy in several tumor types, including urothelial carcinoma of the bladder [47], serous ovarian cancer [46, 48], squamous cell carcinoma [49], pancreatic cancer [50], breast cancer [47], and colorectal cancer [51, 52], and can separate cancer survivors from non-survivors [53]. In some tumor types, Treg accumulation correlates with a better prognosis. For example, in a large series of 967 stage II and stage III CRC, a high density of FoxP3-expressing intra-tumoral Treg was associated with improved survival and showed stronger prognostic significance than CD8+ T and CD45RO+ T cells [54]. A consensus on the marker set and gating strategy used for enumerating Treg in clinical samples has been recently established [55], with CD3, CD4, CD25, CD127, and FoxP3 markers as the minimally required markers to accurately identify human Treg. Furthermore, staining for Ki67 and CD45RA might provide useful information on the activation status of this cell population. The marker set was validated using PBMC from cancer patients as well as cells from TDLN and fresh tumor samples. A phenotyping panel that is not limited by the constraints of intracellular staining has been proposed by Roederer [56], and it considers Treg markers of activation and suppression. Other activation markers, such as CD39, CTLA-4, LAP, GARP, PD-1, and PD-L1, should be included in the monitoring of Treg for cancer patients as surrogate markers for Treg function and potentially eliminating the need for Treg isolation and in vitro suppression assays [57].

During the expansion phase that follows administration of blinatumomab, a bispecific CD3 and CD19 antibody, to patients with B cell precursor acute lymphoblastic leukemia, bone marrow-infiltrating T cells express a skewed T cell receptor (TCR) repertoire compared with peripheral blood T cells suggesting that clonal expansion occurred within the TME and might affect clinical outcome [58]. Massive parallel sequencing can be used to characterize the complete immune repertoire of patients. ImmunoSEQ (Adaptive Biotechnologies, Seattle, WA) offers a proprietary suite of high-throughput immune profiling assays and powerful online software. Multiplex PCR primers target all possible combinations of the noncontiguous (Vβ), diversity (Dβ), and joining (Jβ) gene segments of the β chain locus. The result of such an assay is millions of sequences per sample – and a quantitative description of the immune cell populations [59].

#### Myeloid cells

Tumor-associated macrophages (TAM) comprise up to 50% of malignant tumors. Due to their plasticity, it has been challenging to measure and classify these cells. TAM can be reprogrammed to type 2 macrophages (M2) by microenvironmental factors, as a result of alternative activation by Th2-biased cytokines, such as IL-10 [60]. M2 macrophages can be identified based on their expression of CD163 (scavenger receptor) and CD206 (mannose receptor) coupled with traditional monocyte markers such as CD14, HLA-DR, and CD11b. Although tumor infiltration with TAM has been demonstrated to correlate with poor clinical outcome, recent studies have suggested that high TAM densities could also be predictive of better patient survival as shown in prostate cancer [61]. Thus there is an urgent need to harmonize the phenotypic studies to accurately discriminate M1 from M2 macrophages and to correlate the density of macrophage populations with clinical outcome following immunotherapy [62].

Myeloid-derived suppressor cells (MDSC) consist of immature myeloid progenitor cells with the ability to suppress proliferation and effector functions of T cells [62, 63]. MDSC are expanded in patients with a variety of tumors. In contrast to murine MDSC, the markers used for identification of human MDSC subpopulations are still under discussion. In human PBMC, monocytic and granulocytic MDSC exhibit a CD11b+HLA-DR^neg/low^CD14+CD15- and CD11b+CD14-CD15+ or CD11b+CD14-CD66b+ phenotype, respectively [64]. While monocytic MDSC express the myeloid marker CD33, granulocytic MDSC display CD33^dim^ staining. Furthermore, HLA-DR-CD33+ cells contain mixed groups of MDSC comprising more immature progenitors. It has been proposed that HLA-DR-CD33+ cells be defined as ‘early-stage’ MDSC [64]. A study analyzing the efficacy of multi-peptide vaccination in patients with renal cell carcinoma (RCC) showed that two out of six phenotypically-defined MDSC populations were of prognostic value for overall patients’ survival [65].

#### Monocytes and Antibody-Dependent Cellular Cytotoxicity

Intriguingly, in a study of 29 patients with advanced cutaneous melanoma treated with ipilimumab it was shown that ipilimumab can engage ex vivo CD16-expressing, non-classical monocytes (CD14+CD16++), resulting in antibody-dependent cellular cytotoxicity-mediated lysis of Treg [66]. Patients responding to ipilimumab display significantly higher frequencies of non-classical monocytes at baseline compared with non-responder patients.

The diagnostic potential of intermediate CD14++CD16+ monocytes has also been shown in patients with CRC [67]. Intermediate monocytes were significantly elevated in these patients, with the highest frequencies detected in those with localized disease. The frequency of CD14+CD16+ monocytes was negatively associated with tumor size and pathological stage in patients with breast cancer [68]. The expansion of intermediate monocytes could be driven by monocyte chemoattractant protein-1 (MCP-1), which may be inhibited by the addition of neutralizing antibodies against MCP-1 to the monocyte cultures [68]. Finally, vaccination of patients with stage IV melanoma using Melan-A/MART-1:26-35(27 L) and gp100:209-217(210 M) peptides might augment the frequency of CD14+CD16+ monocytes as well as their expression of CD40/CD86 co-stimulatory molecules and antigen-presenting function [69]. Increases in both intra-tumoral and circulating CD14+HLA-DR^low/neg^ monocytes have been correlated with poor prognosis in RCC patients [70].

### Tissue analysis

While less invasive for patients in comparison to tissue biopsies, the degree to which peripheral immune monitoring is appropriate and useful in a given therapeutic context depends upon the treatment modality in question. Measures of peripheral antigen-specific T cells elicited by vaccines or persisting in the circulation following the administration of T cell based therapies have shown correlations with clinical outcome in some studies [71, 72], but not in others [73–75]. In the context of immune checkpoint blockade, anti-CTLA-4-directed agents have been suggested to expand the breadth of antitumor immunity through peripheral T cell priming [76], whereas PD-1/PD-L1-directed therapy is thought to predominantly act at the level of the TME in tumors with a pre-existing T cell infiltrate [77]. Therefore, the ability to detect pharmacodynamic changes in peripheral blood lymphocyte populations or soluble serum factors that are relevant for treatment outcome may differ between these agents and across various immunotherapies in general. More broadly, a comprehensive and integrated biosampling approach of tissue and peripheral blood in the clinical setting as well as in preclinical models may allow the identification of less invasive biomarkers that reflect clinically meaningful aspects of the immune TME, which will improve the triage and management of patients in the era of cancer immunotherapy. Overall, new tissue analysis tools, rigorous validation, and standardization of methods will help us understand better the dynamic nature of immune-tumor interaction [78].

#### Tissue collection and variability

Significant hurdles exist to the use of archival, fresh tumor biopsies, and TDLN samples for correlative studies. In particular, sample quantity, variability in sample handling and processing, and tissue heterogeneity may all impact the pre-analytical variability of tumor-based immune assays. The volume of tumor tissue routinely obtained in diagnostic biopsies is often limiting for the purposes of high-dimensionality immune monitoring and necessitates a rigorous assessment of assay requirements and prioritization of sample workflow. Moreover, the quality of such routinely obtained tissues may be highly variable. Core or needle biopsies taken from different parts of a tumor mass may manifest significant differences in tumor, stromal, and immune cell composition. For surgical or excisional samples, warm and cold ischemia time is a critical parameter impacting the suitability of the tissue for use in complex immunoassays. Similar considerations apply for the handling of core or needle biopsy samples. Procedures for tissue collection, formalin fixation, and paraffin embedding remain non-standardized across institutions, while standardized protocols for other forms of tissue disposition (freezing, preservation in a nucleic acid stabilization medium such as RNAlater™, direct fresh tissue handling) are often not in place.

Despite these limitations, significant insights into tumor immunobiology have been made using archival formalin-fixed, paraffin-embedded (FFPE) samples. However, working with such samples requires rigorous characterization of pre-analytical variability as it impacts the intended set of assays, followed by hypothesis testing in an appropriately sized dataset that takes into account the anticipated pre-analytical variability. Analytical variability may further compound data quality and interpretation, particularly as it impacts the ability to make comparisons across different studies (this is addressed in greater detail by Working Group 1). For example, differences in sensitivity and specificity of detection antibodies for immunohistochemistry (IHC) or flow cytometry, the qualitative and quantitative scoring algorithms (e.g., analysis of whole tissue sections vs fields of view in IHC), and different method-based reaction principles (e.g., NanoString based gene expression profiling [79] and full RNAseq), represent only some parameters that will complicate direct data comparison.

As patients are exposed to an ever-increasing repertoire of immunotherapies and other anticancer agents, archival tissue, mostly originating from primary diagnostic biopsies, is less likely to be representative of the immune microenvironment at the time of disease progression or relapse. In these cases, fresh tumor biopsies are warranted to characterize tumor immune status at relapse/progression. More generally, because of the factors cited above that impact the pre-analytical variability of archival tissue, dedicated research biopsies taken in the context of detailed SOP for sample acquisition, annotation, handling, and disposition are preferable to archival tumor specimens whenever feasible, acceptable for study design, and ethically appropriate. Dedicated research personnel should be utilized and given responsibility for tissue acquisition and transport, physician training, and other aspects of sample acquisition and handling. Moreover, surgeons, interventional radiologists, and others obtaining samples should be properly trained and tightly integrated into the research team. Inclusion of patients as well as clinical personnel in the scientific discussion, when feasible, will increase patient willingness to donate tissue specimens and ultimately result in better sample quality. Further, proper annotation of research samples is critical to document the anatomic site (preferably including sub-localization within a given lesion), as well as parameters related to tissue collection and handling, such as time from biopsy/excision to fixation (warm and cold ischemia time), and freezing or other storage/processing steps. Where feasible, samples should be annotated with data related to the location of the lesion on radiographic imaging to allow for appropriate data interpretation in the respective context and the longitudinal resampling of the same anatomic site.

#### Multi-institutional studies

Special considerations are necessary when performing tumor immune monitoring in the context of multi-institutional studies. Quality control measures and analytical approaches should be put in place to both minimize and quantify site-dependent variability. This can include centralized specimen shipping kit preparation, overnight specimen shipment in temperature controlled containers, and processing upon arrival. For example, standardized approaches to sample processing, fixation and embedding (or alternate tissue preparation approaches appropriate for a given protocol), as well as sample storage and shipping should be used. It is desirable to centralize as many analytical steps as possible, including tissue sectioning and preparation (e.g., nucleic acid extraction) and analytical assay work. Samples received from multiple institutions should be analyzed in batches, and batched (or real-time) analysis should be used to support the early detection of pre-analytical or analytical quality control issues to ensure that these sources of variation are minimized. Any potentially problematic samples should be annotated accordingly to flag them.

#### Other sources for variability

Pre-analytical variability is influenced not only by technical factors, but also by biological heterogeneity. Where such heterogeneity cannot be fully controlled, it must be well characterized in order to guide the proper design of hypothesis-driven translational research studies [80]. Intra-tumoral heterogeneity of tumor cell clonotypes has been clearly documented through the observation of distinct somatic mutation profiles at different regions within a single lesion [81, 82]. Clonotypic heterogeneity between primary and metastatic lesions and from one metastasis to the next has also been well documented and can directly translate to the heterogeneity of clinical response between lesions within a single patient, which impacts overall disease outcome and treatment opportunities [6, 7]. Likewise, the immune TME may exhibit inter- and intra-lesional heterogeneity. For example, PD-L1 expression has been observed to be discordant between tumor sites in some cases [83]. Preliminary data also show significant intra-patient, inter-lesional diversity in TCR clonality and immune gene expression.

Experimentally, such biological heterogeneity can be controlled for in several ways. At the most basic level, routine morphologic characterization by hematoxylin and eosin (H&E) or cytokeratin staining is critical for confirming the histology of each sample, and SOP that utilize these data to determine sample adequacy and uniformity should be utilized. Where possible two or more cores should be evaluated; multiple cores should be combined for technical approaches that do not preserve geospatial data (e.g., gene expression profiling, whole exome sequencing, TCR deep sequencing, bulk proteomics), in order to compensate for intra-tumor heterogeneity. Where more uniform sampling of specific cell populations is desired, laser capture microdissection can be utilized for cell isolation.

#### Early insights into the TME and immunotherapy

Regardless of these analytical challenges, significant insights have been made into the biology of the tumor immune microenvironment by direct interrogation of tumor tissue. In particular, methodologies for interrogating the tumor-immune interface have demonstrated both prognostic and predictive values in the setting of checkpoint blockade immunotherapy. As discussed elsewhere, both detailed measures of tumor immune infiltration (the immune contexture) and more streamlined biomarkers representing the same phenomenon (the immunoscore) have shown prognostic value in CRC that may exceed that of traditional TNM staging [44, 84]. These data show that spatial distribution of immune cell infiltrates within the TME will be as important as quantitative assessment toward understanding the underlying biology.

Despite the documented geospatial and temporal heterogeneity of PD-L1 expression, PD-L1 on both tumor cells and immune-infiltrating cells has been shown to be a sensitive and specific biomarker of response to PD-1/PD-L1 directed therapies in tumor types such as non-small cell lung cancer (NSCLC) and urothelial carcinoma [85, 86]. Such insights have been made using archival FFPE samples, often of variable age relative to time of study enrollment. More recently, additional biomarkers have demonstrated predictive value in the setting of CTLA-4 or PD-1-directed immunotherapy, although the performance characteristics of these biomarkers have not yet been fully elucidated. These include measures of non-synonymous mutational load and predicted neoepitopes [87–90], TCR clonal diversity [91], apposition of PD-1 and PD-L1 on adjacent T cells and tumor/stromal cells [91], direct correlation of mutational tumor load and TIL infiltration [92], and expression of cytotoxicity or IFN-γ-associated genes characteristic of a T cell inflamed microenvironment [89, 93].

These data indicate that properly controlled tissue acquisition and analysis, as well as the use of complementary and reinforcing technologies (e.g., orthogonal characterization of biomarkers by multiplex IHC [94] and gene expression profiling), could overcome issues related to pre-analytical and analytical variability, allowing for reproducible assessment of clinically meaningful biomarkers in the TME. Furthermore, additional development and investment in such technologies will allow the use of more effective combinations of tissue analysis tools with limited material that is available in a clinical setting.

### Bone marrow

Evaluation of anti-tumor immunity in hematologic malignancies should include an evaluation of the bone marrow [17]. Indeed, several studies have shown that properties of T cells or other immune cells within the bone marrow of patients with hematologic malignancies may differ considerably from those in the circulation [95–97]. Evaluation of the bone marrow is typically performed with a bone marrow aspirate as well as a bone marrow (trephine) biopsy. Below, we describe some of the key considerations when analyzing immune responses in the bone marrow.

#### Collection and adequacy of the specimen

Technical considerations for ensuring the collection of adequate specimen are perhaps the most important element for quality control. It is therefore essential that the aspirate be analyzed at the bedside for adequacy of the specimen per the International Council for Standardization in Hematology (ICSH) guidelines [98]. Large volume aspirates from a single site may simply lead to dilution from peripheral blood and should be avoided. Use of needle redirects to access different regions of the bone through a single skin puncture may be useful but still carry the risk of aspiration from a hemorrhagic site. When possible, we recommend obtaining a paired blood sample so that the phenotypic and functional aspects of blood versus marrow mononuclear cells may be directly compared. For example, in contrast to the peripheral blood, T cells in the human marrow are mostly memory T cells and are enriched for CD8+ T cells [99]. Bone marrow samples collected in sodium heparin are sufficient for most immune monitoring assays wherein analysis by flow cytometry or mass cytometry is the focus [99]. However, EDTA may be the preferred anticoagulant in some instances, particularly when concurrent PCR-based molecular studies are desired. When quantitative changes in immune cell populations during immunotherapy are considered important, it is recommended that the sample dedicated for immune monitoring should be the first sample from the collection site [100]. In contrast to the clinical diagnostic samples which typically get prioritized, this often requires a needle redirect. In addition to the aspirate, collection and evaluation of the biopsy specimen is essential to gain insights into the location of immune cells and cell-cell interactions. ICSH guidelines recommend that at least 2 cm cores should be obtained. In settings wherein the amount of aspirate is inadequate, we suggest routinely obtaining touch preparations of the marrow biopsies. Finally, we strongly recommend that immune monitoring protocols for the marrow (at least those intended towards discovery of new targets) routinely include the preparation of “particle clots” using published guidelines [98, 101]. This is because processing of marrow biopsies typically involves decalcification protocols, which cause nucleic acid or protein damage and impact staining for several antigens. Clot sections do not require decalcification. Another approach is to consider snap-freezing a small (e.g., 0.5 cm) portion of the core, which can subsequently be utilized for analysis of gene expression and downstream deconvolution of data [102].

#### Specimen transport and initial processing

As with peripheral blood, marrow aspirates can be safely transported overnight at room temperature to reference laboratories, and such transport protocols have been successfully utilized in large multicenter studies involving correlative studies on bone marrow specimens [103]. Transport on ice or at lower temperatures leads to loss of mononuclear cell yield. Marrow aspirates also seem to have a greater tendency to clot than blood samples, and it is therefore essential to ensure adequacy of anticoagulant in the tube. Trephine core biopsies are typically added to the fixative at bedside and may be fixed using several different methods. A standard fixative is neutral buffered formalin. Fixation times vary between 1 and 24 h, but are typically 4–6 h. We strongly recommend using a pre-specified fixation time for all specimens in a clinical trial. Fixation longer than 24 h may negatively impact antigen retrieval and should be avoided.

#### Further processing and downstream applications

In contrast to other tissues, isolation of mononuclear cells from the bone marrow does not require enzymatic digestion steps. However, for samples with particulate appearance, we recommend initial dilution of the aspirate in sample buffer and use of a 0.1 micron filter to remove particulate/bone fragment debris. Ficoll density gradient centrifugation remains the most common approach for the isolation of mononuclear cells from the bone marrow. Mononuclear cells isolated from the marrow aspirate have been successfully utilized for a range of downstream immune monitoring assays including flow cytometry based assays, ELISPOT, MHC tetramers, mass cytometry, TCR sequencing as well as genome wide analyses of sorted cells [99, 100, 104, 105].

Trephine biopsies also require decalcification, which can be achieved by several methods. Decalcification with EDTA results in better preservation of nucleic acids but is slower than other acid reagents [98]. The combination of neutral buffered saline fixation followed by EDTA decalcification is the current format preferred by most investigators, as it provides adequate morphology, preserves nucleic acids for molecular studies, and antigens for IHC.

### Microbiome

The analysis of the microbiome is not yet routinely part of the evaluation of immunity in cancer patients and in immunotherapy trials; however, emerging evidence of the important role of the microbiome in modulating anti-cancer immunity and the effectiveness of different types of cancer therapy suggests that this analysis could provide important information regarding the immune status of the patients and their ability to respond to therapy. Biomarkers could be identified and the microbiome could possibly be targeted to improve therapeutic response.

#### The microbiome modulates cancer initiation, progression and response to therapy

Similar to all mammalian organisms, the epithelial barrier surfaces in the human body are colonized by commensal microorganisms (the microbiome) with the largest microbial mass present in the lower intestine [106]. Thus, we are meta-organisms, or symbionts, in which our host cells and the microbial cells cohabit and interact with each other [107, 108]. By regulating human physiology and, in particular, inflammation and immunity, the presence and composition of the microbiome can affect cancer initiation, progression, and response to therapy [109–111]. Viruses and bacterial species have been implicated in oncogenesis [112]. Infection with one bacterial species, *Helicobacter pylori*, has been clearly associated with stomach cancer, and it is recognized as a class 1 human carcinogen [113]. However, several bacterial species have been described that are likely to be involved in the initiation and progression of other cancers such as CRC and gallbladder cancer [109, 114]. In addition, the composition of the microbiome at the epithelial barriers may affect the progression of tumors in sterile tissues not directly colonized by the microbiome [115]. The microbiome composition in cancer patients may be altered due to the presence of the tumor and to a larger extent due to the effect of therapeutic treatments. Use of antibiotics, radiation, and chemotherapy treatments induce persistent changes in the composition of the microbiome, often associated with a reduction in the number of bacterial species present. Following allogeneic bone marrow transplantation, the diversity of the intestinal microbiome at engraftment is an independent predictor of mortality, with higher diversity predicting a more favorable outcome [116]. Recently, experimental evidence as well as initial data in patients have shown that the efficacy of anti-cancer therapy, including adoptive T cell transfer after total body irradiation, immunostimulating oligonucleotides, chemotherapy with cyclophosphamide and platinum compounds as well as immune checkpoint inhibitors, requires the presence of the intestinal microbiome and is affected by the microbial composition [15, 16, 117–119]. The anti-cancer mechanisms of these therapies rely on the ability of the gut microbiome to educate infiltrating immune cells that produce inflammatory mediators required for the direct antitumor effects of therapy and promote the generation of an anti-tumor adaptive immune response [120]. Several microbial genera or species that promote or antagonize the effect of different types of cancer therapy or the anti-tumor host immune response have been identified.

The study of the composition of the microbial communities in the stool or at other anatomical sites of cancer patients before and after therapy could provide information about the immune status of the patients and contribute to the identification of future biomarkers for prediction of disease progression and response to therapy. The presently available information has been largely obtained in experimental animals, so it will be necessary to collect a wide range of information from clinical studies before being able to evaluate the prognostic significance of the findings and the identification of biomarkers. However, this type of analysis has great potential to provide clinically significant information. In addition, there has been important progress in the development of new methodologies to modify the composition of the microbiome, suggesting the possibility that the microbiome could be targeted to slow tumor progression, prevent cancer co-morbidities, enhance cancer therapy efficacy, and to attenuate treatment toxicity.

#### Development of microbiome studies

Until recently, the study of microbes in human samples relied on labor-intensive microbiology techniques for growing and collecting individual isolates, the data from which were influenced by cultivation conditions. These methods did not allow for complete profiling of the microbial communities present in the samples; however, the advent of next generation DNA sequencing methods has advanced microbial investigations. The most common approach for microbiome studies is the amplification and sequencing of variable regions in the bacterial genes encoding 16S ribosomal RNA to determine the taxonomic composition of the microbiome by comparing them to existing databases. Thus, 16S rRNA gene sequencing permits a more comprehensive assessment of the bacterial communities present in a clinical sample. When incorporating microbiome approaches to cancer studies, each element of a microbiome study is critically important [121].

#### Collection of specimens

The collection of specimens and metadata significantly influences the ability to derive clinically relevant downstream analyses. Many factors are important to consider, including determining the body site(s) of interest, e.g., stool, skin, oral mucosa, vaginal mucosa; selection of cases and controls; frequency of sampling; and method of collection. Because sites with little spatial separation can harbor distinct bacterial communities, consistency in sample collection is important [122, 123]. Predominant bacterial taxa and the microbial biomass are body site-dependent and location identity will determine the methods for collecting specimens and relevant metadata [124]. Most oncology studies that have included microbiome approaches have focused on stool [15, 118, 119]. While various stool sampling and storage methods have been studied, feasibility (e.g., accessibility to storage freezers) and patient participation (e.g., self-collection) may guide selection of optimal collection methods [125–127]. Depending on the clinical study, tumor type, and/or therapeutic intervention, the other commonly studied body sites of skin, oral mucosa, and vaginal mucosa may provide distinct and informative microbiome data. Identifying appropriate controls for oncology patients may be challenging; alternatively, repeat samplings of the same patients can provide internal controls, particularly if specimens are collected both prior to and after a clinical intervention.

#### Sequencing and analysis

Given the potential sources of variation in microbiome studies, standardization is crucial for study quality and reproducibility [128]. After collection and processing of specimens, regions of bacterial 16S ribosomal RNA gene are amplified and sequenced. Because newer sequencing platforms do not sequence the complete 16S rRNA genes, only some of the variable regions can be selected and sequenced, which allows effective identification of the genera present but can reduce the ability to identify bacteria at the species level. Primer selection is usually based on the source of the clinical biospecimens, e.g., V4 primers for stool samples and V1-3 primers for skin samples, to optimize species-level identification of sequences [129]. Different platforms can be used for amplicon sequencing. At present, the Illumina’s MiSeq is the most commonly used.

Various pipelines and tools are available to facilitate analyses of amplicon sequencing data. Most popular are Quantitative Insights Into Microbial Ecology [130] and mothur [131]. Alternatively, all genes in all microorganisms in a given sample can be analyzed by shotgun metagenomic sequencing. Metagenomic analysis allows identification not only of bacteria, fungi, and viruses present in a sample but also which genes and gene functions are present in the community. More recently, metatranscriptome analysis has been used to sequence the RNA in a sample and to evaluate which genes are transcribed, and to what extent. Metagenomic and metatranscriptomic analyses require a much higher depth of sequencing (and higher costs) to obtain sufficient coverage of the different microorganisms, and the bioinformatics analysis of the data is more challenging than for amplicon sequencing [132].

## Immune monitoring assays

### Antigen-specific T cells

Peptide-MHC microarrays and other multimeric technologies have been developed as high-throughput technologies for the evaluation of antigen-specific T cell responses [133, 134]. Peptide-MHC multimers tagged with unique DNA barcodes have been recently used for multi-parallel screening of >1000 T cell specificities in complex cellular suspensions [135]. Biotinylated DNA barcodes and peptide-MHC molecules are attached to a PE-labelled dextran backbone carrying streptavidin. MHC multimers-binding T cells can be sorted based on the PE label. DNA barcodes are amplified and sequenced, and the relative numbers of DNA barcode reads is used to determine the composition of antigen-responding T cells in a single sample. This technology has allowed for the identification of melanoma-associated T cell specificities in two melanoma samples directly after enzymatic digest, where the number of TIL was 18,000 and 48,000, respectively [135]. T cell populations were detected in the frequency range of 20-0.01% of CD8+ T cells. Furthermore, specificity profiling was corroborated by assessing functional responsiveness by intracellular cytokine staining upon virus and cancer target recognition. The use of DNA barcode-labelled MHC multimers also enabled the detection of neoepitope-specific T cell populations in cancer patients directly from peripheral blood, with important implications for immune monitoring studies.

### Cytometry by Time-Of-Flight (CyTOF)

Mass cytometry is a fusion of two experimental platforms, i.e., flow cytometry and elemental mass spectrometry, and was initially developed to increase the number of cellular parameters that could be quantified simultaneously [136, 137]. Rather than coupling probes (often antibodies) to fluorophores, mass cytometry experiments utilize probes chelated to unique stable, heavy-metal isotopes, such as the lanthanide series metal ions, which bind targets of interest on and/or within the cell, enabling the attached metal ions to serve as reporters for the expression level of up to 40 targets [136]. Efforts are currently ongoing to harmonize individual mass cytometers’ performance to a common standard of signal intensities and detection limits [138]. Mass cytometry has been recently used to dissect the human mucosal immune system in health and disease, allowing the identification of 142 immune subsets with tissue and disease specificity [139]. This technology is expected to impact immune monitoring strategies and to accelerate the development of individualized therapeutics.

### High-throughput proteome-based technologies

Antibodies detected in the serum of tumor patients can help to identify tumor-associated antigens (TAA) as potential markers for early diagnosis of cancer, for prognosis, for prediction of therapy response as well as for identification of therapeutic targets [140]. To facilitate autoantibody discovery, several different strategies have been developed to simultaneously identify multiple antibodies. Technologies currently available for serologic analyses include SEREX (serological identification of antigens by recombinant expression cloning), phage display, SERPA (serological proteome analysis)/PROTEOMEX (proteomics combined with SEREX), different protein arrays, SomaScan, and MAPPing [141].

#### SEREX

SEREX has been developed primarily for the determination of humoral immunity to TAA by using tumor cDNA libraries in lambda vectors expressed in *E. coli*, which are then transferred to nitrocellulose membranes and incubated with sera from cancer patients and respective control donors. The clones reactive to sera are identified by sequencing [142].

Using this method >1000 TAA have been identified, including NY-ESO-1, which was discovered from an esophageal cancer cDNA library. Next to the use in human patients and clinical trials, SEREX has been also employed in murine transgenic models to predict TAA. Furthermore, a sera database has been established, which is for public access and allows the addition of data from other centers. However, one major limitation of this technology is the failure to detect post-translational modifications.

#### PROTEOMEX/SERPA

In addition to SEREX, PROTEOMEX also termed SERPA was developed using two-dimensional polyacrylamide gel electrophoresis (2D-PAGE) followed by Western blot analysis of the gels followed by their incubation with sera of patients and healthy volunteers [143, 144]. In addition, two-dimensional immune affinity chromatography followed by proteolysis and mass spectrometry has been used to identify novel TAA or respective biomarkers. Although these tools are very robust, the disadvantages of these proteome-based technologies are their labor intensity with limitations in sample capacity, while the mapping could be automated in the future.

#### Protein arrays

Other proteome-based high-throughput analyses include automated protein microarrays of serum antibodies from cancer patients versus healthy controls. With this technology, a large series of proteins can be evaluated, which are either derived from cDNA or peptide phage display libraries [145]. By using protein arrays with a known panel of proteins, an induction of antibody responses against TAA has been recently demonstrated in a study using ipilimumab associated with GM-CSF treatment [146]. The development of antibodies to NY-ESO-1 post-treatment was identified in one clinical responder and one non-responder, suggesting that immunotherapy can induce immune responses to other known TAA. Other sources for immune genomic arrays apart from libraries include recombinant proteins or tumor lysates. The implementations of recombinant proteins for the array are multiple, but also more costly and may not account for post-translational modifications like SEREX. In order to take into account the effects of post-translational modifications on epitope recognition with respect to aberrant glycosylation of the tumor protein, high-throughput analysis using a glycopeptide discovery platform for proteomics profiling has been developed [147]. Although this glycopeptide platform allows high-throughput analyses, it has yet to be validated in particular regarding reproducibility and stability of this technique.

#### SomaScan

Aptamer-based protein array monitoring has recently become available. A particular form of modified aptamers with slow off-rate (SOMAmers) allows for the comparative evaluation of proteins in as low as 70 μl of serum or plasma (or other biological fluids). The SOMAscan assay is highly multiplexed, sensitive and quantitative. This assay is based on the use of a new generation of protein-capture SOMAmer reagent [148]. Native proteins contained in biological samples are captured by SOMAmers immobilized on streptavidin-agarose beads via a photo-cleavable biotin linker. Unbound proteins are washed away. The proteins captured are then biotinylated, the complex biotinylated protein/SOMAmer is released from the capture beads by the UV-induced photocleavage of the photosensitive linker. Magnetic-streptavidin beads capture the freed protein-SOMAmer complexes while the SOMAmers that did not bind to a protein are washed away. Captured SOMAmer-protein complexes are then denatured and the SOMAmers (each containing a unique 40-nucleotide tag) are hybridized onto a high-density array of complementary probes. The hybridized SOMAmers are detected on a DNA array reader, which quantifies the presence of each SOMAmer using classic DNA detection methods. By transforming each individual protein concentration into a corresponding SOMAmer reagent concentration, the SOMAscan assay is not limited by variation between lots of protein standards. The SOMAscan assay measures over 1300 protein analytes that cover a diverse set of molecular functions. Targets to date extensively cover major gene families including receptors, kinases, growth factors, and hormones, and span a diverse collection of secreted proteins, including cytokines and their soluble receptors, and intracellular and extracellular proteins or domains. The assay covers a wide concentration range by using a systematic dilution scheme based on the normal abundance of the protein measured. The analysis of the SOMAscan is performed using classic DNA array data analysis and is based on bioinformatics tools that have been developed for gene array analysis.

#### Multiplexed ELISA-type assays

Chemokines and cytokines are small molecules, which play an important role in an array of physiologic, but also pathophysiologic acute (e.g. infections) and chronic (e.g. cancer) immune responses. Therefore the measurement of chemokines and cytokines can be used to monitor the immune system, as the composition of these small molecules yields insights into the immune cell repertoire and functions both in the disease state as well as in response to immunotherapy [149]. In addition, soluble adhesion molecules and MMP are also suitable biomarkers for clinical trials [22].

Historically, the assessment of cytokines and chemokines has been performed with the single-plex ELISA. Despite the accuracy and value of this technology, it has a limited scope, since determination of the cytokine network interaction is precluded. Furthermore, this method is costly, time consuming, requires a relatively large sample size, and can only measure one analyte per sample. Multiplex immunoassays measuring multiple biomarkers have since been developed and represent an important tool to monitor immune responses [150]. Using the luminex technology, it is possible to evaluate >100 cytokines/chemokines simultaneously with a minimal amount (approximately 50 μl) of sample thus avoiding sample pooling [151, 152]. For the implementation of this assay in clinical trials it is important to determine the accuracy and reliability (including potential antibody cross-reactivity) of the detection method for each analyte. Furthermore pre-analytical variables, such as the anti-coagulant used for the collection of blood, sample preparation, time and temperature storage of samples as well as gender and age of the donor also have an impact on the cytokine/chemokine measurement using luminex and ELISA [152–154]. However, no single method of specimen preparation was clearly superior for the measurement of cytokines. Although there exist anti-coagulant-dependent differences in analyte concentrations, the relative concentrations of the various analytes remain similar for a given anti-coagulant [154].

### Transcriptomics

Gene expression profiles reflect the systemic immune milieu and can be used for immune monitoring purposes as well as to identify predictive biomarkers. In melanoma patients treated with tremelimumab, an IgG2 antibody that targets CTLA-4 on T cells, a genomic signature predictive of prolonged survival has been recently identified, consisting of four gene transcripts [155]. Pretreatment gene expression signatures have also been identified in patients with melanoma and NSCLC receiving MAGE-A3 immunotherapy. Eighty-four genes were identified, in which expression correlated with better clinical outcome [156]. The genes identified were mainly immune-related, including IFN-α and γ pathways and specific chemokines, highlighting the concept that pretreatment gene expression patterns can influence the TME and the patient’s clinical response. The transcriptional profiles of sentinel node biopsies from melanoma patients suggest that infiltration with CD30+ lymphocytes positively associates with disease progression [157].

Microarrays have been very useful for the high-throughput analysis of gene and miRNA expression, but they are limited by the requirement for the use of relatively large quantities of high quality RNA. Next-generation sequencing (NGS) can be used for high-throughput gene expression analysis, but this technology remains costly and data analysis is difficult. Microarrays and NGS have been important discovery tools since they measure the entire transcriptome; however, their use for most immunotherapies is generally restricted to assessing the expression of sets of genes targeted to cells, tumors or pathways of interest, thus representing an opportunity to further take advantage of these powerful tools in the discovery and assessment of biomarkers [158].

Quantitative PCR (qPCR) provides a more accurate measurement of gene expression than microarrays and requires less RNA, but the analysis of the expression of multiple genes with classical qPCR is difficult. Nanofluidics has been used with PCR to make multiplex PCR less labor intensive and less costly. Nanofluidic instruments are available which allow for high-throughput multiplex PCR analysis. One instrument, the BioMark™ system (Fluidigm Corporation) allows for the simultaneous performance of 48 or 96 PCR assays on 48 or 96 samples [159]. This platform can be employed for the analysis of both gene and miRNA expression and has been implemented to measure the expression of more than 90 genes or miRNAs [160, 161]. Digital PCR can also be used for high-throughput high-precision analysis, but multiplex PCR is more difficult. Digital PCR can be performed on chips or in droplets [162, 163].

Molecular “bar coding” is being used for the high-throughput analysis of the expression of multiple genes. The nCounter Analysis System (NanoString Technologies, Inc.) can measure RNA levels of more than 700 genes. Requiring no amplification step, it directly measures low quantities of mRNA using molecular bar codes and digital molecular imaging [79].

### Genome mutation analysis

Somatic mutation can play a critical role in cancer development and progression. Tumor genotyping is important for classifying tumors and predicting response to directed therapies. SNP and other mutations can be detected by fluorescence in situ hybridization (FISH), PCR with sequence-specific primers or probes and Sanger sequencing. These methods are limited by the need for relatively large quantities of DNA and they are relatively slow and expensive, especially when analyzing for multiple mutations [164].

Whole genome or exon sequencing using NGS platforms can be used to analyze the entire genome, but this is not yet practical for routine clinical analysis because of the high cost and large amount of data analysis required. Targeted NGS reduces data analysis requirements and is used for the targeted analysis of mutations in cancer genes. The targeted sequences can be isolated using sequence-specific primers or probes and multiple loci can be targeted [165]. Nanofluidic platforms and PCR have also been used with NGS to analyze multiple loci [166]. Customized microarrays can also be used for targeted SNP analysis (GeneChip Custom SNP Kits, Affymetrix).

## Analysis of the systemic host response

The systemic assessment of immune regulation and modulation can quickly result in a morass of data that spans patients, time points, assays, tissues, and organizations. For example, tissues sampled from a given patient might include PBMC, serum, tumor biopsies, and TDLN and these might be assayed by a combination of flow or CyTOF (cytometry by time-of-flight) phenotyping, phospho-flow, Luminex or protein arrays, and gene expression. Organizational considerations might include multiple cores at the same or different institutions, and academic, government, and industry participants from multiple countries. Consequently, the analysis of such multifaceted data may be fragmented by assay or organization in ways that undermine measurement of the systemic response. To increase the value of these expensive and complex data sets, the data must be merged into a consistent assay-agnostic format that spans assays, tissues, and organizations. This integrated heterogeneous data set can be referred to as a “het set.”

The het set offers several advantages, the first of which is that it supports the goals of capturing and characterizing the systemic host response. A het set also provides a common technical and conceptual representation of an otherwise unwieldy data set and the same analytical tools and techniques can be applied to hundreds or thousands of analytes from multiple assays. Finally, established multivariable analytical approaches can be applied to the integrated whole, with an emphasis on results that span assays or tissues. Table 1 provides a small extract from a representative het set in a “long” format, with a single data point occupying each row. It should also be noted that data from different assays might require processing or normalization prior to inclusion in the het set [57].

**Table 1:** Sample extract from a representative integrated heterogeneous data set (het set)

Person	Day	Tissue	Assay	Analyte	Readout	Units
1–52	0	PBMC	Flow phenotyping	CD4+ Treg	3.2	% of parent
1–52	0	Tumor	Flow phenotyping	CD4+ Treg	5.1	% of parent
1–52	0	Serum	Luminex	IL2	3.8	pg/ml
1–52	1	Serum	Luminex	IL2	2.7	pg/ml
1–52	5	Serum	Luminex	IL2	2.5	pg/ml
1–52	0	Whole blood RNA	Gene expression	IL2	10.1	log normalized expression

Once a het set has been created, a variety of well-established analytical principles and techniques can be considered [167]; novel analytical approaches are not necessarily needed to obtain novel scientific findings or to improve patient care. A common example of an analytical goal that can be supported by a het set is the identification of biomarkers that distinguish responders from non-responders. This is considered a classification problem, which is fundamentally different than looking for analytes that are statistically different between responders and non-responders. This scenario calls for a “supervised” algorithm, in which we know the answer (response, non-response) and are looking for a set of analytes that help us arrive at that answer. A decision tree is one such supervised approach. Alternatively, if one is looking for a variety of patterns in the data that help us to better understand the relationships between patient characteristics and analytes, then an “unsupervised” approach, in which there is not a specific answer is appropriate. Hierarchical clustering and association rule mining are examples of unsupervised approaches. Ideally, the analytical approaches will provide both quantitative and visual results. Another consideration is whether the analytical techniques are magnitude-insensitive, that is, able to easily support data from assays yielding wildly different numeric ranges. Furthermore, the results suggested by any analysis should be vetted for biological relevance and replicated in independent data sets or studies. The following five techniques, detailed below, can provide insight into the systemic host response and are applicable to het sets: regression modeling, network of cross-compartment correlations, penalized regression, decision tress, and association rule mining.

*Regression modeling* supports both simple models (such as response α β_1_ x analyte) and more complex models (such as response α β_1_ x analyte + β_2_ x treatment + β_3_ x sex + β_4_ x age). In both simple and complex models, the β terms are the estimated coefficients or contributions of the predictor variables to the outcome variable. Complex multivariable models can be longitudinal models or time-to-event (survival) models and account for variables like treatment type, sex, and age. Longitudinal models may be particularly appropriate for characterizing immune response over time and can account for patient-specific trends. Response can be categorical (responder versus non-responder) or continuous (progression-free survival). A strategy that is common in gene expression analysis is to build such a model for all genes and focus on a handful with the smallest p-values on the coefficient of interest. While it is fast and easily understood, this approach does not provide a comprehensive picture that accounts for systemic responses or for correlations amongst analytes.

One approach to building a systemic *network of cross-compartment correlations* is to start with a regression model in which one analyte is the outcome and another is the predictor, e.g., assayA.analyte1 ~ β_1_ x assayB.analyte2 + β_2_ x response. As with multivariable regression, a variety of other predictors can be included in the model. Once the model results for all possible pairs of analytes are obtained, the results can be filtered to pairs of analytes from different assays or tissues and have reasonably small p-values on effects of interest, such as both the correlation between the analytes, and the effect of the response. Given 50 to 100 of such correlations, the relationships across the analytes can be tallied and the networks of correlations can be visualized. For example, Whiting et al. identified a network of 61 highly correlated analytes spanning flow phenotyping, phospho-flow, and serum proteins as measured by Luminex, after accounting for age, sex, and cytomegalovirus status. Of these, 9 analytes were connected to at least 7 other analytes [168]. This approach provides the flexibility of a regression-modeling framework, while accounting for all possible pairwise correlations between analytes and filters allow for cross-assay or cross-tissue correlations. Additional approaches to network analysis are reviewed by Wang and Huang [169].

A *penalized regression* approach, such as lasso or elastic-net [170, 171], selects a subset of variables that best predict outcome, in part by constraining a function of the sum of the regression coefficients, and the outcome can be categorical or numerical. Penalized regression has been used by researchers to predict SLN11 levels in breast cancer patients [172], to predict post-treatment levels of CD137+ NK cells in various cancers [173], and to model progression-free survival as a function of serum cytokines [174]. One advantage of this regression approach is that it performs both feature selection and model building in a single pass. A limitation of this approach is that all analytes are normalized prior to model building, and numeric results are expressed in terms of standard deviations from the mean of any particular analyte. This can complicate both interpretation and application to subsequent data sets. Essentially, we have to assume that the mean and standard deviation of any particular analyte in our working data set are comparable to that in a replication set.

*Decision trees* are a supervised machine learning technique for classification. The algorithm interrogates all analytes to find the one that best splits the observations into categorical outcomes such as responder and non-responder. Then, it interrogates all remaining analytes to find the next best split, and so on, until a series of splits yields relatively pure groups. Advantages of decision trees include ease of interpretation, support for both continuous and categorical attributes, and support for analytes of a variety of scales. Furthermore, they can be particularly useful when data is bimodal*—*for example, very high Treg and very low Treg. O’Donoghue et al. used a decision tree on gene expression to classify good and poor prognosis in dogs diagnosed with canine osteosarcoma [175]. *Random forests* are an extension of decision trees, in which hundreds or thousands of trees are built from randomly selected subsets of both analytes and patients. Patients are then classified based on their most common assignment across all of the trees. Researchers have used this approach to identify serum proteins that can stage prostate cancer patients [176]. Random forests have the advantage of being more robust to data outliers. The method has also been extended to support time-to-event (survival) data [177]. However, the resulting model is not easily visualized since it includes many trees.

*Association rule mining* is an unsupervised machine learning technique for pattern identification. Since it works only on categorical data, continuous data must be first converted to categorical data. Such conversions can be quantile-based (e.g. quartile) or based on reference ranges (below, within, above) [178]. Association rules yield “if-then” statements such as “If Ki67 expression is low and IHC score = 2, then the HER2:CEP17 ratio (as measured by HER2 FISH pharmDx) is negative (less than 2:2),” reported in a study of breast cancer patients [179]. Association rules can be quantified by the percentage of the study population to which they apply, and the percentage of the time that they are true. As with the pairwise regression models discussed above, association rules can be filtered for those that span assays or tissues.

These are only a few of the many approaches available for analyzing multivariable multi-assay data sets. Others include principle component analysis, hierarchical clustering, and artificial neural networks. Given a het set that includes data from multiple assays, time points and tissues, the systemic host response can indeed be analyzed in an assay-agnostic manner.

## Clinical application of immune monitoring

### Approach to monitoring immunotherapy for GI malignancies

Immune-based treatment approaches have revolutionized oncology in recent years. Various treatment strategies have received US Food and Drug Administration (FDA) approval including cell vaccination for prostate cancer as well as immune checkpoint inhibition targeting the CTLA-4 or the PD-1/PD-L1 axis in melanoma, lung, and kidney cancer. Additionally, cell based therapies (adoptive T cell therapy, chimeric antigen receptor [CAR] T cells and, TCR transduced T cells) have demonstrated substantial efficacy in patients with B cell malignancies and melanoma. Immune checkpoint inhibitors in particular have generated enormous excitement across the entire field of oncology, providing a significant benefit to a minority of patients as well as teaching us a great deal about the immune system in our efforts to predict who will benefit from treatment. However, with some notable exceptions, most studies in patients with tumors of the GI tract using this type of treatment approach have been disappointing. One of the first studies demonstrating impressive results of PD-1/PD-L1-directed therapy was discouraging from a GI cancer viewpoint [180]. There were no responses in any of cohorts containing patients with colorectal (*N* = 18), pancreatic (*N* = 14), and gastric (*N* = 7) cancer. Similarly, negative results for GI cancers were seen in other studies of both anti-PD-1 and anti-CTLA-4 therapy [181–183].

#### Mismatch repair deficiency and anti-tumor immunity

One notable exception to this disappointing preliminary experience has been in mismatch repair-deficient CRC where significant responses to PD-1 pathway inhibition have been observed [184]. The defective mismatch repair system results in a marked increase in the non-synonymous mutagenic burden within tumors, increasing the likelihood that a tumor-specific neoantigen, capable of recognition by the immune system, is generated [185]. This is certainly relevant for any tumor type that happens to have a high mutagenic burden because of either inherited or acquired mismatch repair deficiency – resulting in a degree of microsatellite instability (MSI) – or other factors. Various tumors of the GI tract have been shown to occur in patients with inherited mismatch repair deficiency. MSI is present in 10–20% of sporadic colorectal [186], gastric [186], and ampullary cancers [187]. Between 0.3 and 13% of pancreatic cancers are reported to have MSI as well [188] and recently a small proportion (5.9%) of biliary cancer have been identified to have a high mutational load [189]. Consequently, immune monitoring has become important for GI malignancies (Table 2).

**Table 2:** Monitoring immunotherapy for GI malignancies

Marker	Specimen	Use
MSI	Tumor	Determine eligibility for anti-PD1 treatment
Quant HCV	Serum	Response to anti-CTLA-4 treatment in patients with HCV infection
Pathology	Liver biopsy	Rule out drug induced hepatitis
ALT/AST	Serum	Liver toxicity
Gut microbiome	Stool	Response to treatment with immune checkpoint inhibitors

#### Anti-viral responses as surrogate markers for an active immunotherapy

A number of GI cancer types are typical inflammation associated tumors. Almost 90% of all patients with hepatocellular carcinoma (HCC) also show an underlying liver disease. Chronic viral hepatitis (hepatitis B virus [HBV] and hepatitis C virus [HCV]) is a major risk factor for the development of liver cirrhosis and HCC. Immune checkpoint inhibitors are currently being evaluated in HCC patients with underlying chronic HBV and HCV infection. Interestingly, not only did tremelimumab show early signs of anti-tumor efficacy, but it also induced a decrease in HCV viral load from 3.78 x 10^5^ IU/ml at day 0 to 1.69 x 10^3^ IU/ml. In parallel, the investigators observed a general trend to increased number of virus-specific IFN-γ-producing lymphocytes post-treatment [38]. We have observed similar effects in HCC patients with chronic HBV or HCV infection [190]. In summary, anti-viral responses can be used to track the effect of those approaches aiming to enhance antigen-specific T cell immunity.

#### Liver toxicity

Immune-stimulatory mAbs are currently being evaluated as antitumor agents. Although overall toxicity from immunotherapy drugs such as anti-CTLA-4, anti-PD-L1/PD-1, and anti-CD40 appears to be moderate, liver toxicities have been reported and are not completely understood.

Transient dose-related elevations in serum liver transaminases and total bilirubin were observed after infusion of anti-CD40 [191]. It was thought that this effect was due to CD40+ hepatocytes, which underwent apoptosis upon CD40 activation. We have been able to show that agonistic CD40 antibody caused liver damage within 24 h after injection in a variety of different murine tumor models. Here, liver damage was induced by the generation of reactive oxygen species produced by intrahepatic myeloid cells, which accumulate in the liver of tumor-bearing individuals [192]. Therefore, liver toxicity may be mediated by anti-CD40 activated intrahepatic myeloid cells rather than a direct effect of anti-CD40 on hepatocytes [193]. Transient transaminitis has also been observed in patients with HCC treated with tremelimumab [38]. A remarkable rise in serum transaminases was observed after the first dose in more than half of the patients. However, it was not associated with a parallel decline in liver function and did not recur in the following treatment cycles.

This observation was unexpected since inflammatory hepatic adverse events (AE) related to anti-CTLA-4 were uncommon in clinical studies. Of any grade, these AE were reported in 3.8% (5/131) of patients treated with ipilimumab monotherapy at 3 mg/kg in a phase III trial [194]. Kleiner and Berman studied 5 patients in which a liver biopsy was taken to rule out drug-induced autoimmune hepatitis.

The histologic changes observed with ipilimumab-related hepatitis were similar to those with acute viral and autoimmune hepatitis and it was not possible to make a definite diagnosis of a drug-induced hepatitis. Hepatic inflammation in the five patients reported resolved with appropriate immuno-suppressive therapy, and the authors suggest that patients who receive immune checkpoint inhibitor therapy should be monitored at regular intervals for biochemical and pathological evidence of hepatitis so that appropriate treatment can be promptly administered [195].

#### Endoscopy

Patients undergoing immune checkpoint therapy could develop enterocolitis as an adverse event [195]. A common side effect in such instances is diarrhea, which warrants endoscopic procedures such as upper endoscopy and colonoscopy. It should be noted that these tests can also be used to obtain tumor biopsies and monitor progress during the course of treatment.

### Biomarkers and cell therapies

#### Characteristics of adoptively transferred cells associated with better clinical outcomes

The adoptive transfer of TIL for the treatment of patients with metastatic melanoma has produced promising clinical results. More favorable clinical results have been associated with greater in vivo persistence of infused TIL one month after therapy [196]. Characteristics of TIL that correlate with more favorable outcomes include longer telomeres and the administration of larger numbers of TIL, CD8+ cells, and CD8+CD27+ T cells [196, 197]. TIL that spend less time in culture, so-called “young” TIL, have a phenotype consistent with an earlier differentiation state including longer telomeres and higher levels of CD27 and CD28 expression [198–200] and these cells may be more effective clinically [197]. Analysis of TIL cells obtained from patients with metastatic melanoma has shown that reduced expression of the chemokine receptors CXCR3 and CCR5 and the presence of the CCR5-Δ32 polymorphisms, which encodes a protein that is not expressed, were associated with better response to TIL therapy [201].

Preclinical models have shown that the phenotype of adoptively transferred T cells can influence their effectiveness. Antigen-specific central memory T (T_CM_) cells are more effective for adoptive T cell therapy than effector memory T cells; transferred T_CM_ survive longer in vivo [202]. Adoptively transferred memory T cells that have stem cell-like qualities, stem memory T cells (T_SCM_), result in greater in vivo expansion, longer persistence and better anti-tumor activity [203, 204]. T_SCM_ are characterized as CD45RA+, CD62L+, CCR7+, and CD95+. Some investigators are developing methods to enrich adoptively transferred T cells with T_SCM_ or T_CM_ characteristics [205].

Clinical studies of adoptively transferred T cells engineered to express CAR have found that the in vivo expansion of these cells also been associated with favorable clinical outcomes [206]. When CD19 CAR T cells are used to treat children and young adults with acute lymphoblastic leukemia, the transferred T cells can expand several-fold. Peak expansion of CD19 CAR T cells occurred at 14 days post-infusion and the cells persisted up to two years [206, 207]. Peak expansion was associated with disappearance of circulating leukemic blasts in responding patients. Patients responding to the therapy had higher levels of circulating CD19 CAR T cells than those that did not respond [206].

#### Tumor-trafficking potential of adoptively infused T cells

The trafficking of effector T cells to tumor sites is a prerequisite for their antitumor activity. Tumor irradiation has been shown to shape a pro-inflammatory microenvironment that permits the extravasation of T cells and promotes their effector function [208].

CD19-targeted T cells may be more rapidly cleared from the circulation in the presence of a higher peripheral blood tumor burden, likely as a result of tumor infiltration and disappearance from the circulation [209]. However, in a patient with chronic lymphocytic leukemia who died 44 h after CAR T cell infusion, staining of autopsy tissues with anti-CAR antibodies showed rapid T cell trafficking to tumor sites, including lymph nodes, bone marrow, and liver [209]. Studies of autologous anti-LeY CAR immunotherapy in patients with acute myeloid leukemia have shown migration of the adoptively infused T cells to the bone marrow and the skin, as well as persistence for up to 10 months [210].

CAR T cells containing the CD28 endodomain may be endowed with enhanced expansion potential and persistence compared with CAR T cells lacking this endodomain [211]. The analysis of skin biopsies from a patient with non-Hodgkin lymphoma showed that 20% of the gated CD3+ lymphocytes co-expressed the CAR. This study clearly demonstrates that one of the incremental benefits of incorporating critical costimulatory components into CARs is the ability of T cells to infiltrate and mediate anti-tumor effects in tissues.

The tumor trafficking potential of activated T cells bearing a CAR specific for the tumor antigen GD2 can be enhanced by forced co-expression of the chemokine receptor CCR2b, which directs migration towards CCL2, a chemokine produced by several tumors. This strategy translated into improved homing (>10 fold) to CCL2-secreting neuroblastoma compared to CCR2-negative T cells, as well as greater in vivo anti-tumor activity [212]. Forced expression of CCR4 by effector T cells has been shown to enhance their migration to the Reed-Sternberg cells of Hodgkin lymphoma (HL) [213], which predominantly produce TARC/CCL17 and MDC/CCL22. Furthermore, T cells expressing both CCR4 and the HL-associated antigen CD30 manifested greater cytotoxic function and in vitro cytokine secretion, and mediated better tumor control in mice engrafted with human HL [213].

#### Monitoring the levels of adoptively transferred T cells

Monitoring the circulating levels of adoptively transferred TIL and lymphocytes engineered to express CAR or high affinity TCR is important for improving the effectiveness of these therapies. The survival of T cells can be monitored by labeling a fraction of the cells with radionuclides such as chromium-51 or indium-111, however radiolabeling requires dedicated space, highly trained staff, and it is not widely available. The intravascular persistence of T cell clones prepared from TIL can be measured by TCR-specific PCR [214]. T cell clone persistence can be monitored by amplification of the TCR beta-chain region gene and the relative expression of the TCRBV gene products can be determined using a panel of monoclonal antibodies and flow cytometry [215]. This method has been used to show that the degree of persistence in the peripheral blood of adoptively transferred T cell clones was associated with melanoma regression [215]. Another study found that the persistence for one month of adoptively transferred T cell clones prepared from TIL was associated with clinical responses [196]. While this technique has provided important insights, it is limited by the need to isolate and characterize clones. In addition, the quantitative ability of this assay is limited.

Monitoring the levels of CAR T cells in the peripheral blood is easier and has yielded important information. The percent of T cells expressing CARs can be measured using flow cytometry. If the scFV region of the monoclonal antibody used in the CAR is of mouse origin, then goat antibodies directed to mouse F(ab)2 can be used to quantitate CAR T cells. To detect CD19 CAR T cells by flow cytometry, anti-Fab antibody staining and labeled CD19 protein have been used [216]. Flow cytometry using anti-idiotype monoclonal antibody has been used to detect CD19 CAR T cells derived from CD19 mouse monoclonal antibody clone FMC63, [206, 217, 218]. CAR T cell expansion can also be detected by quantitative qPCR [219, 220].

#### Cytokine release following cell infusion

The rapid expansion of adoptively transferred CD19 CAR T cells and the disappearance of leukemic cells is associated with clinical toxicity due to cytokine storm [221]. Cytokine release syndrome (CRS) is a non-antigen specific toxicity that occurs as a result of high levels of activation of lymphocytes or myeloid cells. It is associated with elevated circulating levels of several cytokines including IL-6, IFN-γ, and TNF-α. Clinically, patients with CRS may experience fever, tachycardia, and hypotension. It can result in cardiac dysfunction, adult respiratory distress syndrome, renal failure, hepatic failure, or neurotoxicity [221]. It is more likely to occur in patients with higher tumor burdens and greater T cell expansion [207, 221]. IL-6 appears to play an important role in the pathogenesis of CRS and the anti-IL-6 receptor antibody, tocilizumab, is often an effective therapy. The clinical use of tocilizumab has also been explored in patients with acute lymphoblastic leukemia who develop CRS after blinatumomab immunotherapy [222].

CRP has been found to be an effective biomarker for CRS [221]. CRP is an acute phase reactant produced by the liver. Its production is largely dependent on IL-6. In patients with ALL treated with CAR T cells, CRP levels have been found to be associated with the IL-6 levels and CRS severity [206].

## Conclusions and recommendations

The field of immune monitoring has helped advance immunotherapy for cancer. All clinical trials of immune therapies for cancer should include a structured plan for sample collection, biomarker analysis, and data analysis. Sample collection and analysis must be adopted for each study, but several points should be considered (Table 3).

**Table 3:** Type of sample and high throughput assessments

Sample Type	Suggested High-Throughput Assessment
Serum/plasma	• Luminex • Protein arrays • SEREX • PROTEOMEX/SERPA • SomaScan
PBMC	• Flow cytometry • Phospho-flow cytometry • MHC multiplexed multimers • nCounter Analysis System (NanoString) • qPCR • Gene expression microarrays • NGS • miRNA expression analysis • ImmunoSEQ
Tissue	• Multiplexed IHC or immunofluorescence • Flow cytometry • qPCR • Gene expression microarrays • NGS • miRNA expression analysis

- Due to the complexity and our current limited understanding of the underlying biology of cancer immunotherapies, routine, direct evaluation of tumor samples, archival as well as fresh paired tumor samples and direct comparison to peripheral samples, should be considered as a high priority.
- In addition to analyzing plasma, serum, and peripheral blood leukocytes, consideration should be given to the analysis of tissue samples, the microbiome and, if appropriate, adoptively transferred immune cells.
- Mutiplexed, high throughput assessment allows for the analysis of multi-analyte signatures which can lead to a better understating of key mechanisms and the identification of biomarkers.
- Analysis might include flow cytometry, high throughput proteomics, mRNA, miRNA, and DNA mutagenous assays (Table 3).
- Computation biologists should be enlisted to best assess the systemic immune response for expertise in combining data across platforms correctly (Table 1).

## Abbreviations

2D-PAGE: Two-dimensional polyacrylamide gel electrophoresis
ACK: Ammonium chloride potassium
AE: Adverse event(s)
ALC: Absolute lymphocyte count
CAR: Chimeric antigen receptor(s)
CRC: Colorectal cancer
CRP: C-reactive protein
CRS: Cytokine release syndrome
CyTOF: Cytometry by time of flight
EDTA: Ethylenediaminetetraacetic acid
ELISA: Enzyme-linked immunosorbent assay
EV: Extracellular vesicle(s)
FDA: US Food and Drug Administration
FFPE: Formalin-fixed, paraffin-embedded
FISH: Florescence in situ hybridization
GI: Gastrointestinal
H&E: Hematoxylin and eosin
HBV: Hepatitis B virus
HCC: Hepatocellular carcinoma
HCV: Hepatitis C virus
HL: Hodgkin lymphoma
ICSH: International Council for Standardization in Hematology
Ig: Immunoglobulin
IHC: Immunohistochemistry
M2: Type 2 macrophages
MCP-1: Monocyte chemoattractant protein-1
MDSC: Myeloid-derived suppressor cells
miRNA: MicroRNA
MMP: Matrix metalloproteinase(s)
MSI: Microsatellite instability
NGS: Next-generation sequencing
NK: Natural killer
NSCLC: Non-small cell lung cancer
PBMC: Peripheral blood mononuclear cell(s)
PBS: Phosphate buffered saline
PROTEOMEX: Proteomics combined with SEREX
qPCR: Quantitative polymerase chain reaction
RCC: Renal cell carcinoma
SEREX: Serological identification of antigens by recombinant expression cloning
SERPA: Serological proteome analysis
SNP: Single nucleotide polymorphism(s)
SOMAmer: Slow off-rate modified aptamer
SOP: Standard operating procedure(s)
TAA: Tumor-associated antigen(s)
TAM: Tumor-associated macrophage(s)
T_CM_: Central memory T cell(s)
TCR: T cell receptor
TDLN: Tumor draining lymph node(s)
TIL: Tumor infiltrating lymphocyte(s)
TME: Tumor microenvironment
Treg: Regulatory T cell(s)
T_SCM_: Stem memory T cell(s)
WG: Working group
